# Association of Prenatal and Postnatal Exposures to Warm or Cold Air Temperatures With Lung Function in Young Infants

**DOI:** 10.1001/jamanetworkopen.2023.3376

**Published:** 2023-03-17

**Authors:** Ariane Guilbert, Ian Hough, Emie Seyve, Matthieu Rolland, Joane Quentin, Rémy Slama, Sarah Lyon-Caen, Itai Kloog, Sam Bayat, Valérie Siroux, Johanna Lepeule

**Affiliations:** 1 Team of Environmental Epidemiology Applied to Development and Respiratory Health, Institute for Advanced Biosciences, Université Grenoble Alpes, INSERM, CNRS, La Tronche, France; 2 Institute of Environmental Geosciences, Université Grenoble Alpes, Saint Martin D’Hères, France; 3 Université de Paris Cité, INSERM, INRAE, Center of Research in Epidemiology and Statistics, Paris, France; 4 Pediatric Department, Grenoble Alpes University Hospital, La Tronche, France; 5 Department of Geography and Environmental Development, Ben-Gurion University of the Negev, Be’er Sheva, Israel; 6 Lung Function Laboratory, Grenoble Alpes University Hospital, La Tronche, France

## Abstract

**Importance:**

Little is known about long-term associations of early-life exposure to extreme temperatures with child health and lung function.

**Objectives:**

To investigate the association of prenatal and postnatal heat or cold exposure with newborn lung function and identify windows of susceptibility.

**Design, Setting, and Participants:**

This population-based cohort study (SEPAGES) recruited pregnant women in France between July 8, 2014, and July 24, 2017. Data on temperature exposure, lung function, and covariates were available from 343 mother-child dyads. Data analysis was performed from January 1, 2021, to December 31, 2021.

**Exposures:**

Mean, SD, minimum, and maximum temperatures at the mother-child’s residence, estimated using a state-of-the-art spatiotemporally resolved model.

**Main Outcomes and Measures:**

Outcome measures were tidal breathing analysis and nitrogen multiple-breath washout test measured at 2 months of age. Adjusted associations between both long-term (35 gestational weeks and first 4 weeks after delivery) and short-term (7 days before lung function test) exposure to ambient temperature and newborn lung function were analyzed using distributed lag nonlinear models.

**Results:**

A total of 343 mother-child pairs were included in the analyses (median [IQR] maternal age at conception, 32 [30.0-35.2] years; 183 [53%] male newborns). A total of 246 mothers and/or fathers (72%) held at least a master’s degree. Among the 160 female newborns (47%), long-term heat exposure (95th vs 50th percentile of mean temperature) was associated with decreased functional residual capacity (−39.7 mL; 95% CI, −68.6 to −10.7 mL for 24 °C vs 12 °C at gestational weeks 20-35 and weeks 0-4 after delivery) and increased respiratory rate (28.0/min; 95% CI, 4.2-51.9/min for 24 °C vs 12 °C at gestational weeks 14-35 and weeks 0-1 after delivery). Long-term cold exposure (5th vs 50th percentile of mean temperature) was associated with lower functional residual capacity (−21.9 mL; 95% CI, −42.4 to −1.3 mL for 1 °C vs 12 °C at gestational weeks 15-29), lower tidal volume (−23.8 mL; 95% CI, −43.1 to −4.4 mL for 1 °C vs 12 °C at gestational weeks 14-35 and weeks 0-4 after delivery), and increased respiratory rate (45.5/min; 95% CI, 10.1-81.0/min for 1 °C vs 12 °C at gestational weeks 6-35 and weeks 0-1 after delivery) in female newborns as well. No consistent association was observed for male newborns or short-term exposure to cold or heat.

**Conclusions and Relevance:**

In this cohort study, long-term heat and cold exposure from the second trimester until 4 weeks after birth was associated with newborn lung volumes, especially among female newborns.

## Key Points

**Question:**

Are mothers’ and newborns’ exposures to heat and cold associated with newborn lung function?

**Findings:**

In this cohort study of 343 mother-child pairs, both mothers’ and newborns’ exposures to heat and cold were significantly associated with decreased functional residual capacity and increased respiratory rate in female newborns. In addition, cold was significantly associated with decreased tidal volume in female newborns.

**Meaning:**

This work suggests an association between ambient temperature and newborns’ respiratory systems and underlines the vulnerability of pregnant women and their future children to climate change.

## Teaser

This cohort study of mother-child dyads explores the associations between prenatal and postnatal temperature exposures and male and female newborn lung function.

## Introduction

Climate change is increasing the intensity and frequency of extreme temperatures,^1^ affecting human health.^2^ Western Europe is a heat-wave hot spot compared with northern midlatitudes.^3^ Such changes challenge the body’s adaptative abilities, especially among pregnant women and newborns.^4,5^

Increasing evidence indicates that maternal exposure to high ambient temperatures and, to a lesser extent, low temperatures could increase the risk of adverse birth outcomes.^6,7,8^ However, there is a paucity of information on the subsequent risks for children’s development. Chronic respiratory diseases are a leading cause of morbidity and mortality worldwide,^9^ and the importance of early lung development on the lifelong lung trajectory, including lung growth and peak lung function in early adulthood, is well established.^10,11^ Current data suggest that unusual ambient temperatures may promote or worsen respiratory diseases, such as asthma, rhinitis, or respiratory tract infections.^12,13^ Decreased forced vital capacity^14^ and peak expiratory flow^15,16,17^ have been associated with short-term and long-term exposure to extreme temperatures in healthy children or children with asthma. During pregnancy, high diurnal temperature variations have been associated with significant increase of pneumonia diagnosis^18^ and the common cold in children.^19^

To date, no study has investigated the association of maternal heat or cold exposure with lung function in newborns. Specific periods of vulnerability to heat during organogenesis and fetal life exist.^20^ In addition, male and female populations show differences in lung growth and airway development,^21,22,23^ lending support to differential responses to environmental stressors.^14,18,19^ This finding paves the way for the investigation of sex-specific critical windows of susceptibility. We leveraged lung function measurements performed in newborns of the SEPAGES (Suivi de l’Exposition à la Pollution Atmosphérique durant la Grossesse et Effets sur la Santé [Assessment of Air Pollution Exposure During Pregnancy and Effect on Health]) mother-child cohort and a cutting-edge spatiotemporal model of ambient temperature exposure combined with distributed lag models to explore the associations between prenatal and postnatal long-term and short-term temperature exposures and male and female newborn lung function.

## Methods

### Study Population

**Figure 1.**
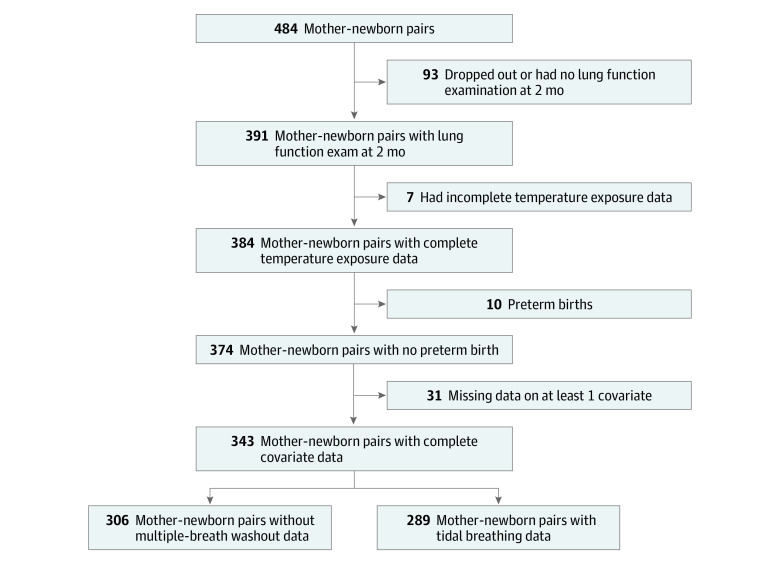
Flowchart of the Study Population

This work relied on the prospective mother-child cohort SEPAGES, which enrolled 484 pregnant women (<19 gestational weeks at inclusion, having a singleton pregnancy) between July 8, 2014, and July 24, 2017,^24^ in the French Grenoble metropolitan area (445 000 inhabitants), a flat valley surrounded by Alpine mountains with a contrasted climate of continental, oceanic, Mediterranean, and mountain influences. This study included 343 mother-child pairs with complete data on temperature exposure, newborn’s lung function, covariates, and no preterm births (Figure 1; eTable 1 in Supplement 1). Informed written consent was obtained from the parents of the expected child before inclusion. The study was approved by the Ethics Committee Sud-Est V and the National Commission on Informatics and Liberty. This study followed the Strengthening the Reporting of Observational Studies in Epidemiology (STROBE) reporting guideline for cohort studies.

### Exposure Assessment

Mother-child’s home addresses at the street number level (including changes during the follow-up period) were geocoded using the BD TOPO database from the National Institute of Geographic and Forest Information (eMethods in Supplement 1). Daily minimum, mean, and maximum ambient temperatures at the home addresses were estimated using a state-of-the-art multiresolution spatiotemporal model covering the continental French territory.^25^ We used data at the 200-m resolution for women living in urban areas (245 women [71%] at conception) and at the 1-km resolution otherwise. Four weekly indicators were calculated: (1) nighttime temperature (average of daily minimum temperature), (2) overall temperature (average of daily mean temperature), (3) daytime temperature (average of daily maximum temperature), and (4) variability (SD of daily mean temperature).

### Newborn Lung Function Measurements

Lung function was assessed in newborns (median [IQR] age, 6.7 [6.3-7.3] weeks) by trained professionals and following the European Respiratory Society/American Thoracic Society guidelines^26^ (eMethods in Supplement 1). Flow-volume curves were measured by recording 10 minutes of tidal breathing in the sleeping child. The mean minute ventilation, tidal volume, respiratory rate (RR), and time to peak tidal expiratory flow to total expiratory time (tPTEF/tE) ratio were assessed.

Lung volume and ventilation heterogeneity were explored by performing 3 measurements of nitrogen multiple-breath washout with pure oxygen. Use of pure oxygen induced a transient hypoventilation^27^ with no systematic pattern. Lung Clearance Index (LCI) and functional residual capacity (FRC) were computed and corrected for the degree of hypoventilation by using the residuals of the mixed linear regression of the degree of hypoventilation on LCI and FRC.^28^

### Covariates

Potential confounders were selected a priori based on literature and a directed acyclic graph (eMethods in Supplement 1). Analyses were adjusted for maternal age at conception, prepregnancy body mass index, parity, mode of delivery, breastfeeding, parents’ rhinitis, highest level of education of the parents, prenatal and/or postnatal tobacco exposure, child sex, child age, child weight and height at the lung function test, season at the lung function test, and Normalized Difference Vegetation Index (NDVI; based on Landsat satellite data, calculated in a 100-m buffer around the home address, during June-August the year of birth^29^).

### Statistical Analysis

The associations between exposure to ambient temperature and newborn lung function measurements (continuous variables) were investigated using distributed lag nonlinear models^30^ (DLNM; eMethods in Supplement 1). For each lung function measurement and each exposure indicator, 2 periods of interest were examined simultaneously: (1) long-term exposure, in weeks, including the first 35 gestational weeks and first 4 weeks after delivery; and (2) short-term exposure, in days, encompassing the 7 days preceding and including the day of the lung function tests.

We hypothesized that the associations between temperature exposure (short and long term) and lung function measurements would be nonlinear and modeled the dose response and lag response using natural cubic splines. The number of *df* was set to 2 for all splines, based on the Akaike information criterion and parsimony. The lag response (β and 95% CI) curves were modeled for heat and cold (95th and 5th temperature percentiles, respectively) exposure compared with the median exposure.

We report both the cumulative risk associated with exposure throughout the duration of a critical window and some illustrations of the risk associated with a single week of exposure. Sensitivity analyses on LCI and FRC were performed excluding newborns with high hypoventilation levels (>75th percentile). Analyses were performed using R software, version 4.0.4 (R Foundation for Statistical Computing) and the dlnm package, version 2.4.7. Data analysis was performed from January 1, 2021, to December 31, 2021.

## Results

### Population Characteristics

This study included 343 mother-child pairs (median [IQR] maternal age at conception, 32 [30.0-35.2] years; 183 [53%] male and 160 [47%] female newborns) (Table). A total of 246 mothers and/or fathers (72%) held at least a master’s degree. Among the newborn population, 251 (73%) were not exposed to tobacco in utero or during the postnatal period.

**Table.** Characteristics of the Study Population^a^

Characteristic	All mother-child pairs (N = 343)	Pairs with male newborns (n = 183)	Pairs with female newborns (n = 160)
Highest level of parental education			
Less than a master’s degree	97 (28)	51 (28)	46 (29)
Master’s degree or more	246 (72)	132 (72)	114 (71)
Parental rhinitis			
No	125 (36)	66 (36)	59 (37)
Yes	218 (64)	117 (64)	101 (63)
Prenatal and/or postnatal tobacco exposure			
No	251 (73)	135 (74)	116 (72)
Yes	92 (27)	48 (26)	44 (28)
Mode of delivery			
Vaginal	291 (85)	155 (85)	136 (85)
Cesarean	52 (15)	28 (15)	24 (15)
Parity			
No child	150 (44)	76 (42)	74 (46)
≥1 Child	193 (56)	107 (58)	86 (54)
Breastfeeding			
Not breastfed at 2 mo	46 (13)	28 (15)	18 (11)
Still breastfed at 2 mo	297 (87)	155 (85)	142 (89)
Season at the lung function test			
Winter	86 (25)	40 (22)	46 (29)
Spring	75 (22)	45 (25)	30 (19)
Summer	74 (22)	40 (22)	34 (21)
Fall	108 (31)	58 (32)	50 (31)
Mother’s age at conception, median (IQR), y	32.3 (30.0-35.2)	32.1 (29.6-35.2)	32.5 (30.3-35.1)
Mother’s BMI before pregnancy, median (IQR)	21.4 (19.7-24.0)	21.6 (19.7-23.7)	21.0 (19.8-24.3)
Child age at the lung function test, median (IQR), wk	6.7 (6.3-7.3)	6.7 (6.3-7.3)	6.7 (6.3-7.4)
Child height at the lung function test, median (IQR), cm	56.3 (55.0-58.0)	57.3 (55.6-58.5)	55.5 (54.3-56.8)
Child weight at the lung function test, median (IQR), kg	4.8 (4.5-5.2)	5.0 (4.6-5.4)	4.6 (4.3-5.0)
Summer NDVI the year of birth, median (IQR)	0.5 (0.3-0.6)	0.5 (0.3-0.6)	0.5 (0.4-0.6)
Nighttime temperature, median (IQR), °C^b^	7.8 (6.0-9.4)	7.7 (6.0-9.5)	8.0 (6.0-9.3)
Overall temperature, median (IQR), °C^b^	12.7 (10.5-14.5)	12.4 (10.4-14.4)	13.1 (10.6-14.6)
Daytime temperature, median (IQR), °C^b^	18.6 (16.1-20.8)	18.3 (16.1-20.6)	18.7 (16.6-21.0)
Variability of mean temperature, median (IQR)^b^	7.2 (6.5-7.8)	7.2 (6.5-7.8)	7.1 (6.6-7.8)
LCI, median (IQR)	7.6 (6.9-8.4)	7.9 (7.3-8.5)	7.2 (6.5-7.8)
FRC, median (IQR), mL	105.7 (94.7-115.0)	107.9 (97.3-117.6)	102.7 (93.0-111.8)
Hypoventilation level (mean maximum decrease in tidal volume), median (IQR), %	42.8 (33.4-52.3)	41.0 (32.0-49.3)	45.0 (36.0-54.2)
Tidal volume, median (IQR), mL	34.4 (29.2-39.1)	36.0 (31.5-40.7)	32.2 (27.7-36.5)
Minute ventilation, median (IQR), mL∙min^−1^	1532.5 (1374.7-1703.4)	1567.2 (1389.2-1718.0)	1486.4 (1329.9-1667.8)
Respiratory rate, median (IQR), /min	44.3 (37.8-52.8)	43.2 (37.5-50.1)	45.7 (38.6-56.4)
tPTEF/tE ratio, median (IQR), %	35.4 (29.2-42.7)	33.5 (28.5-41.4)	36.6 (31.2-43.4)

Abbreviations: BMI, body mass index (calculated as weight in kilograms divided by height in meters squared); FRC, functional residual capacity; LCI, Lung Clearance Index; NDVI, Normalized Difference Vegetation Index.

^a^ Data are presented as number (percentage) of study participants unless otherwise indicated.

^b^ Mean throughout the pregnancy.

**Figure 2.**
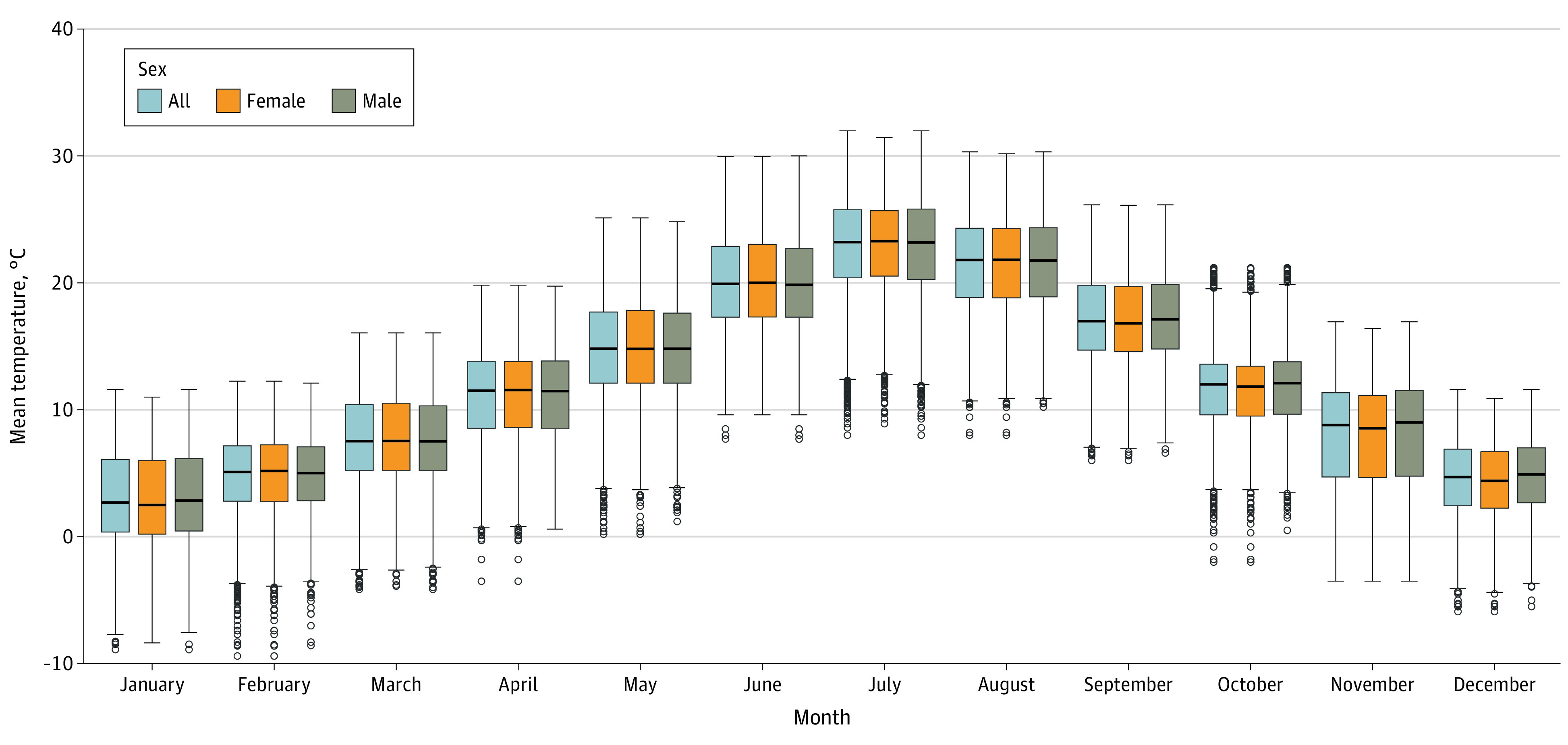
Distribution of Mean Overall Temperatures Aggregated by Month for the First 35 Gestational Weeks and the First 4 Weeks After Birth The bottom and the top of the boxes show the 25th and the 75th percentiles, respectively; the middle line inside the box indicates the median; the whiskers display the minimum and maximum values within 1.5 times the IQR from the first and third quartiles; and the small circles represent the outliers.

The median overall temperature during pregnancy was 12.7 °C (IQR, 10.5 °C-14.5 °C) (Table), with the highest temperatures observed in July (22.8 °C) and the lowest in January (3.0 °C) (Figure 2). The median variability was 7.2 °C (IQR, 6.5 °C-7.8 °C). Temperature exposure was similar across sex.

**Figure 3.**
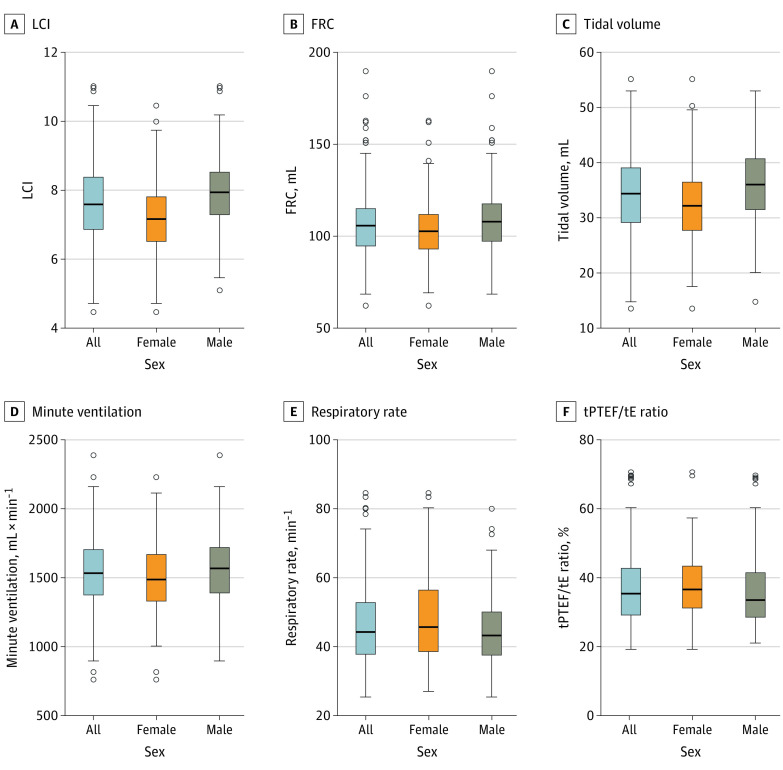
Distribution of Lung Function Measurements at 2 Months The bottom and the top of the boxes show the 25th and the 75th percentiles, respectively; the middle line inside the box indicates the median; the whiskers display the minimum and maximum values within 1.5 times the IQR from the first and third quartiles; and the small circles represent the outliers. FRC indicates functional residual capacity; LCI, Lung Clearance Index; and tPTEF/tE, time to peak tidal expiratory flow to total expiratory time.

Median lung function measures were as follows: LCI, 7.6 (IQR, 6.9-8.4); FRC, 105.7 mL (IQR, 94.7-115.0 mL); tidal volume, 34.4 mL (IQR, 29.2-39.1 mL); minute ventilation, 1532.5 mL∙min^−1^ (IQR, 1374.7-1703.4 mL∙min^−1^); RR, 44.3/min (IQR, 37.8-52.8/min); and tPTEF/tE ratio, 35.4% (IQR, 29.2%-42.7%) (Table and Figure 3). Tidal volume showed moderate to high Spearman correlations with LCI (ρ = 0.58), FRC (ρ = 0.50), and RR (ρ = −0.68). Lung Clearance Index, FRC, tidal volume, and minute ventilation were significantly lower in female than male newborns, whereas RR was significantly lower in male newborns. The studied participants did not significantly differ from those excluded from the analyses, except those excluded were slightly older at the lung function examination (eTable 1 in Supplement 1).

### Ambient Temperature and Newborn Lung Function

#### Heat and Newborn Lung Function

**Figure 4.**
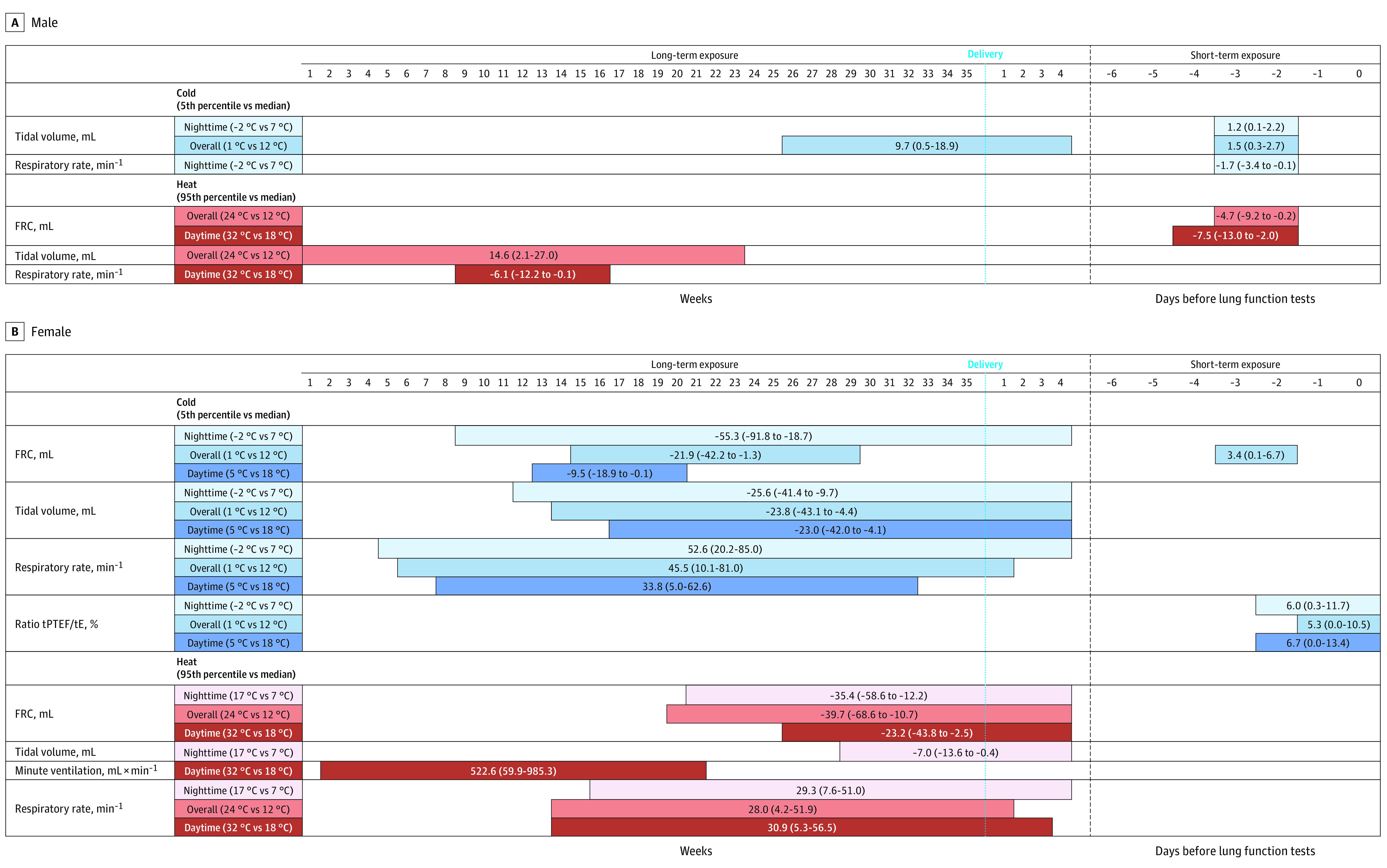
Association Between Cumulative Change in Lung Function Measurements and Long-term and Short-term Exposure to Cold or Heat Bars show the timing of critical windows, with cumulative β (95% CI). Long-term exposure includes the first 35 gestational weeks and the first 4 weeks after delivery; short-term exposure includes the 7 days before the lung function test. Nighttime indicates the average of daily minimum temperature; overall, average of daily mean temperature; daytime, average of daily maximum temperature; and variability, SD of daily mean temperature. Models were adjusted for maternal age at conception, highest level of education of the parents, prepregnancy maternal body mass index, parity, parents’ rhinitis, mode of delivery, breastfeeding, prenatal and/or postnatal tobacco exposure, child sex, child age, child weight and height at the lung function test, season at lung function test, and Normalized Difference Vegetation Index for the year of birth. The number of associations presented varies across sex because the table restricts to the statistically significant windows (ie, significant associations are not consistently observed among heat, cold, and each respiratory measurement for both male and female newborns). The critical window positions can also vary according to sex. FRC indicates functional residual capacity; and tPTEF/tE, time to peak tidal expiratory flow to total expiratory time.

Results obtained from the total population appeared highly driven by sex (eTable 2 in Supplement 1). In female newborns, nighttime, overall, and daytime heat (95th vs 50th temperature percentiles) from the second trimester of pregnancy onward was significantly associated with decreased FRC (Figure 4; eTable 2 and eFigure 1 in Supplement 1). Overall heat during gestational weeks 20 to 35 and weeks 0 to 4 after delivery was related to decreased FRC by 39.7 mL (95% CI, −68.6 to −10.7 mL) compared with the median temperature (24 °C vs 12 °C). Consistent positive associations were also observed for RR for exposure occurring from the second trimester of pregnancy (Figure 4; eTable 2 and eFigure 2 in Supplement 1). Exposure to overall heat during gestational weeks 14 to 35 and the first week of life was linked to increased RR by 28.0/min (95% CI, 4.2-51.9/min) compared with the median temperature. Sporadic associations of long-term exposure to nighttime heat with lower tidal volume and daytime heat with increased minute ventilation were observed.

In male newborns, a significant association between short-term exposure to overall and daytime heat the last 2 to 4 days before the lung function test and reduced FRC was identified (Figure 4; eTable 2 in Supplement 1). Some associations of long-term exposure to overall heat with increased tidal volume and daytime heat with reduced RR were observed.

#### Cold and Newborn Lung Function

In female newborns, long-term exposure to nighttime, overall, and daytime cold (5th vs 50th temperature percentiles) was significantly associated with lower FRC (Figure 4; eTable 2 and eFigure 1 in Supplement 1), with critical windows from the end of the first trimester onward. Overall cold from gestational weeks 15 through 29 was related to decreased FRC by 21.9 mL (95% CI, −42.4 to −1.3 mL) compared with the median temperature (1 °C vs 12 °C). Nighttime, overall, and daytime cold were also significantly associated with decreased tidal volume if they occurred from the second trimester of pregnancy onward. Exposure to overall cold during gestational weeks 14 to 35 and weeks 0 to 4 after delivery was associated with decreased tidal volume by 23.8 mL (95% CI, −43.1 to −4.4 mL) compared with the median temperature. Significant associations were observed for RR, with critical windows starting in the first trimester of pregnancy (eFigure 2 in Supplement 1). The RR increased by 45.5/min (95% CI, 10.1-81.0/min) compared with the median temperature for overall cold exposure throughout gestational weeks 6 to 35 and the first week after delivery. Regarding short-term exposure, nighttime, overall, and daytime cold during the day of the lung function test and the last 2 days before were associated with increased tPTEF/tE ratio. For overall temperature, the estimate reached 5.3 (95% CI, 0.0-10.5) compared with the median temperature. Short-term exposure to low overall temperature was also associated with increased FRC.

Among male newborns, tidal volume tended to be positively associated with short-term exposure (second and third days preceding the lung function test) to nighttime and overall cold (Figure 4; eTable 2 in Supplement 1). We further observed some associations between long-term exposure to overall cold and higher tidal volume as well as between short-term exposure to nighttime cold and lower RR.

#### Temperature Variability and Newborn Lung Function

Among female newborns, exposure to low or high temperature variability, starting toward the end of the first trimester of pregnancy until delivery, was associated with increased LCI and FRC, respectively (eTable 2 in Supplement 1). In male newborns, high temperature variability was associated with increased FRC, minute ventilation, and RR (eTable 2 in Supplement 1).

#### Sensitivity Analyses

Trends were similar when restricting data to newborns with hypoventilation levels below the 75th percentile. Among female newborns, significance of the association observed between long-term exposure to nighttime, overall, and daytime heat and FRC disappeared as well as the one highlighted between long-term exposure to low temperature variability and LCI. Nonetheless, the curves of the lag-response association were similar to the main analyses.

## Discussion

This cohort study found that long-term exposure to both heat and cold was associated with decreased FRC and increased RR in female newborns. Cold was further associated with lower tidal volume in female newborns. For both female and male newborns, long-term exposure to high temperature variability was associated with higher FRC. Unusual temperatures seemed primarily associated with lung volumes and, to a lesser extent, airflows. Critical windows mainly ranged from the second trimester of pregnancy to the first postnatal month.

### Heat and Lung Function

Long-term exposure to heat was significantly associated with decreased FRC and increased RR among female newborns. This convergence of associations based on different lung function tests is interesting. It could potentially be explained by an enhanced respiratory drive elicited by thermoregulatory responses to both high and low temperatures, although these changes should abate on return to thermoneutrality. On the other hand, both associations could be mediated by a reduced lung volume, possibly as a result of slower lung growth due to increased metabolic cost of thermoregulation. The critical windows of susceptibility for FRC and RR extended from the second trimester through the first weeks of life. Sensitivity analyses excluding children with the highest hypoventilation levels showed similar results, although statistical significance was lost. We note that female newborns had slightly higher hypoventilation levels than male newborns and were thus more frequently excluded from the sensitivity analyses. No similar study results exist in children, but among US adults, temperature increase was associated with decreased forced expiratory volume in 1 second,^31^ which seems consistent with our findings. Short-term exposure to heat was associated with decreased FRC among male newborns with 2 to 4 days’ lag. This isolated association should be interpreted with caution. A previous study^16^ found associations with peak expiratory flow and forced vital capacity.

### Cold and Lung Function

Exposure to cold showed similar trends as those observed for heat: significant associations were mainly observed for female newborns, with long-term exposure associated with decreased lung volumes, suggesting restrictive conditions. Long-term exposure to cold was associated with decreased FRC, decreased tidal volume, and increased RR among female newborns. The critical windows of susceptibility were long, ranging from the middle of the first trimester through the first weeks of life. In a national representative sample of school-age children in China, long-term postnatal exposure to low temperatures has been associated with reduced forced vital capacity.^14^

Contrary to a previous study^15^ that found a significant association between short-term exposure to low ambient temperature and decreased peak expiratory flow in healthy Chinese children, no such association was observed in the current study. Short-term exposure to cold was positively associated with female patients’ tPTEF/tE ratio with a lag up to 2 days. Considering that no other respiratory measurement was consistently modified, this result should be considered with caution.

Among male newborns, a trend was observed for short-term exposure to cold and increased tidal volume. However, no other lung function measurement showed similar results, and decreased lung volume after a short-term exposure to cold seems unlikely.

### Temperature Variability and Lung Function

For both male and female newborns, high temperature variability was associated with increased FRC compared with the median temperature variability. In male newborns, this association was accompanied by higher minute ventilation and RR. Such trends might be compatible with airway obstruction.^32,33^ In Chinese children, large temperature variations during the prenatal period have been associated with increased risk of the common cold^19^ and pneumonia.^18^

### Biological Pathways

Thermoregulation is compromised in pregnancy due to increased cardiovascular demands and other physiologic changes.^34^ A wide corpus of animal studies^20,35,36^ showed impaired placental development, increased oxidative stress, inflammatory cytokines, and reduced blood flow to the placenta and uterus after short-term or long-term heat stress exposure. These physiologic alterations may affect lung development of the fetus, in accordance with this study’s results. Direct temperature exposure during the postnatal period could act on the respiratory system as well as on risk factors (eg, allergens) of altered lung function.^12^

Contrary to previous research,^14,18,19,37^ female newborns appeared more sensitive to both cold and heat than male newborns. However, previous studies^23,38^ were performed on older populations and considered different lung function measurements. Data from human and animal studies show different lung growth and airway development in male and female fetuses, linked to sex hormones. Girls often have smaller lungs and alveolar surface areas but larger-caliber airways than boys.^38,39^ In addition, lung surfactant is produced earlier in girls compared with boys, which may induce higher airflow rate and lower airway resistance in girls.^38^

### Strengths and Limitations

This study has several strengths. Innovative early measurements of newborn lung function were performed. Temperature exposure was also assessed at participants’ home address using a cutting-edge spatiotemporally resolved exposure model with excellent performances and showing the highest resolution for France.^25^ Compared with commonly used fixed monitoring stations, this approach better captures fine-scale spatial patterns and identifies urban heat island effects. Lastly, use of DLNM enabled us to analyze the time dimension of the exposure-response association; to consider variation of exposure over time; to identify nonlinear, lagged associations; and to identify specific windows of susceptibility. We considered exposure in the long term, adjusted for both prenatal and postnatal exposure.

This study also has several limitations. This work was based on a relatively small and well-educated population, which might limit the generalizability of the results. However, associations may well be similar or even larger in socially disadvantaged populations, which are additionally expected to be disproportionately exposed to climate change.^40^ From a statistical point of view, numerous tests were performed, leading to possible spurious associations. We balanced this risk by focusing on the most consistent and biologically plausible associations, involving interrelated lung function measurements. Exposure was assessed at participants’ home addresses and restricted to outdoor temperature, which is subject to nondifferential measurement error. In addition, despite correction, measurement error on LCI and FRC due to hypoventilation cannot be completely ruled out. Both exposure and outcome measurement errors may have biased association estimates toward the null. This work should be continued to investigate whether associations observed at 2 months of age translate into formally diagnosed respiratory diseases at older ages or greater susceptibility to respiratory infections.

## Conclusions

This cohort study provides evidence that health consequences of extreme temperatures could be initiated in utero. In a temperate climate, prenatal and postnatal exposure to heat and cold was associated with lung function changes in female newborns. Critical windows of susceptibility ranged from the second trimester of pregnancy until the fourth week of life. Research must continue to better understand the long-term impact of unusual temperatures in early life and to raise awareness of health risks posed by heat and cold exposure during this period among pregnant women, mothers, and health care professionals.

## Notes

Supplement 1. **eMethods.** Supplementary Materials and Methods **eTable 1.** Comparison of the Included and Excluded Participants **eTable 2.** Cumulative Change in Lung Function Measurements Significantly Associated With Exposure to Cold, Heat or Variability of Temperature **eFigure 1.** Lag-Specific Effect of Cold (A) and Heat (B) Compared With the Median Temperature on Functional Residual Capacity for Females **eFigure 2.** Lag-Specific Effect of Cold (A) and Heat (B) Compared With the Median Temperature on Respiratory Rate for Females
eReferences

Supplement 2.
Data Sharing Statement
