# Supplementary material for: Association of Prenatal and Postnatal Exposures to Warm or Cold Air Temperatures With Lung Function in Young Infants
Source: JAMA Netw Open. 2023 Mar 17;6(3):e233376. doi: 10.1001/jamanetworkopen.2023.3376 (PMC10024202; doi:10.1001/jamanetworkopen.2023.3376)
Supplement: Supplement 1. — eMethods. Supplementary Materials and Methods eTable 1. Comparison of the Included and Excluded Participants eTable 2. Cumulative Change in Lung Function Measurements Significantly Associated With Exposure to Cold, Heat or Variability of Temperature eFigure 1. Lag-Specific Effect of Cold (A) and Heat (B) Compared With the Median Temperature on Functional Residual Capacity for Females eFigure 2. Lag-Specific Effect of Cold (A) and Heat (B) Compared With the Median Temperature on Respiratory Rate for Females eReferences [file jamanetwopen-e233376-s001.pdf]

Supplementary Online Content

Guilbert A, Hough I, Seyve E, et al. Association of prenatal and postnatal exposures to warm or cold air temperatures with lung function in young infants. *JAMA Netw Open*. 2023;6(3):e233376. doi:10.1001/jamanetworkopen.2023.3376

eMethods. Supplementary Materials and Methods

eTable 1. Comparison of the Included and Excluded Participants

eTable 2. Cumulative Change in Lung Function Measurements Significantly Associated With Exposure to Cold, Heat or Variability of Temperature

eFigure 1. Lag-Specific Effect of Cold (A) and Heat (B) Compared With the Median Temperature on Functional Residual Capacity for Females

eFigure 2. Lag-Specific Effect of Cold (A) and Heat (B) Compared With the Median Temperature on Respiratory Rate for Females

eReferences

This supplementary material has been provided by the authors to give readers additional information about their work.

© 2023 Guilbert A et al. *JAMA Network Open.*

eMethods. Supplementary Materials and Methods

Exposure assessment

Mother-child's home addresses at the street number level (including changes during the follow-up period) were geocoded using the BD TOPO® database from the National Institute of Geographic and Forest Information. The BD TOPO® is a 3D vector description of the elements of the French territory and its infrastructures, with a metric precision ranging from 1:2000 to 1:50000.

Daily minimum, mean and maximum ambient temperatures at the home addresses were estimated using a state-of-the-art multi-resolution hybrid spatiotemporal model covering the continental French territory[1]. This model combines measurements from weather stations, remotely sensed MODIS land surface temperature and other predictors (e.g. Normalized Difference Vegetation Index (NDVI), elevation, population, land cover, etc.) using linear mixed models, random forest and extreme gradient boosting models. It covers France with a 1km resolution for the whole continental territory and a 200m resolution in urban areas with more than 50 000 inhabitants. The model shows excellent performance with a ten-fold cross-validated $R^2 = 0.97$ and a root mean square error = 1.3°C for temperature mean at a 1km resolution. We used data at the 200m resolution for women living in urban areas (71% of the participants at conception) and at the 1km resolution otherwise.

Four weekly indicators were calculated: 1) night-time temperature (average of daily minimum, Tmin), 2) overall temperature (average of daily mean, Tmean), 3) daytime temperature (average of daily maximum, Tmax) and 4) variability (standard deviation (SD) of daily mean, Tsd).

Newborn lung function assessment

Lung function was assessed in newborns (median: 6.7 weeks; InterQuartile Range [IQR]: 6.3, 7.3) by trained professionals and following the ATS/ERS guidelines[2]. Measurements were carried out during natural sleep, in supine position with the head midline, using an infant face mask (AMBU Size 1 Infant) and excluding from analysis the first 20 to 30 breaths to reach stabilization of the newborn's breathing pattern.

Flow-volume curves were measured by recording ten minutes of tidal breathing in the sleeping child. The mean minute ventilation ($mL.min^{-1}$; total volume of gas which enters or leaves the lung per minute), tidal volume (mL; inhaled air volume per breath), respiratory rate (RR; $breath.min^{-1}$; number of breaths per minute) and time to peak tidal expiratory flow to total expiratory time ratio (tPTEF/tE) were assessed, based on the first 50 regular tidal breaths, excluding sighs and 10 breaths before and after a sigh.

Lung volume and ventilation heterogeneity were explored by performing three measurements of nitrogen multiple-breath washout (N_2MBW) with pure oxygen (O_2), using the Exhalyzer-D© device and Spiroware© software (Ecomedics, Dürnten, Switzerland). For this experiment, the child breathed pure oxygen and the washout of ambient air nitrogen from the respiratory system was followed during quiet sleep tidal breathing. Lung Clearance Index (LCI) and Functional Residual Capacity (FRC; mL) were computed from the recorded N_2 washout curves, while ensuring that tidal volume was within target, no swallowing or sigh occurred in the first 5 breaths and no sign of leak was present. LCI represents the number of lung volume turnovers required to expel N_2 to $1/40^{th}$ (2.5%) of its starting concentration for at least 3 consecutive breaths. It allows to investigate lung ventilation inhomogeneity due to alterations in conducting, small airway's structure and therefore to detect early airway damage. FRC represents the lung volume at the end of a normal expiration. In this study, use of pure oxygen induced a transient hypoventilation characterized by decreased tidal volume[3] with no systematic pattern. LCI and FRC values were corrected for the degree of hypoventilation (maximum drop of tidal volume in the first 15 breaths following oxygen exposure as compared to the mean tidal volume in breaths before oxygen exposure) by using the residuals of the mixed linear regression (to account for repeated measures by subject) of the degree of hypoventilation on LCI and FRC, adjusted for child gestational age, sex, weight and height at the lung function assessment[4].

Covariates

Data on other covariates were mainly collected through questionnaires and clinical examination.

Potential confounders were selected a priori based on literature and a directed acyclic graph (cf. Figure below). Analyses were adjusted for maternal age at conception, prepregnancy BMI, parity, mode of delivery, breastfeeding, parents' rhinitis, highest level of education of the parents, pre- and/or postnatal tobacco exposure, child sex, child age, weight and height at the lung function test, season at the lung function test, Natural Difference Vegetation Index (NDVI; based on Landsat satellite data, calculated in a 100m buffer around the home address, during June-August the year of birth[5]).

Analyses were not adjusted for gestational age at birth, newborn respiratory infections because they may represent mediators between pre- and postnatal exposure to heat, cold and newborn lung function. Despite its beneficial role in lung development[6], prenatal vitamin D intake was not included as a covariate due to a lack of reliable information.

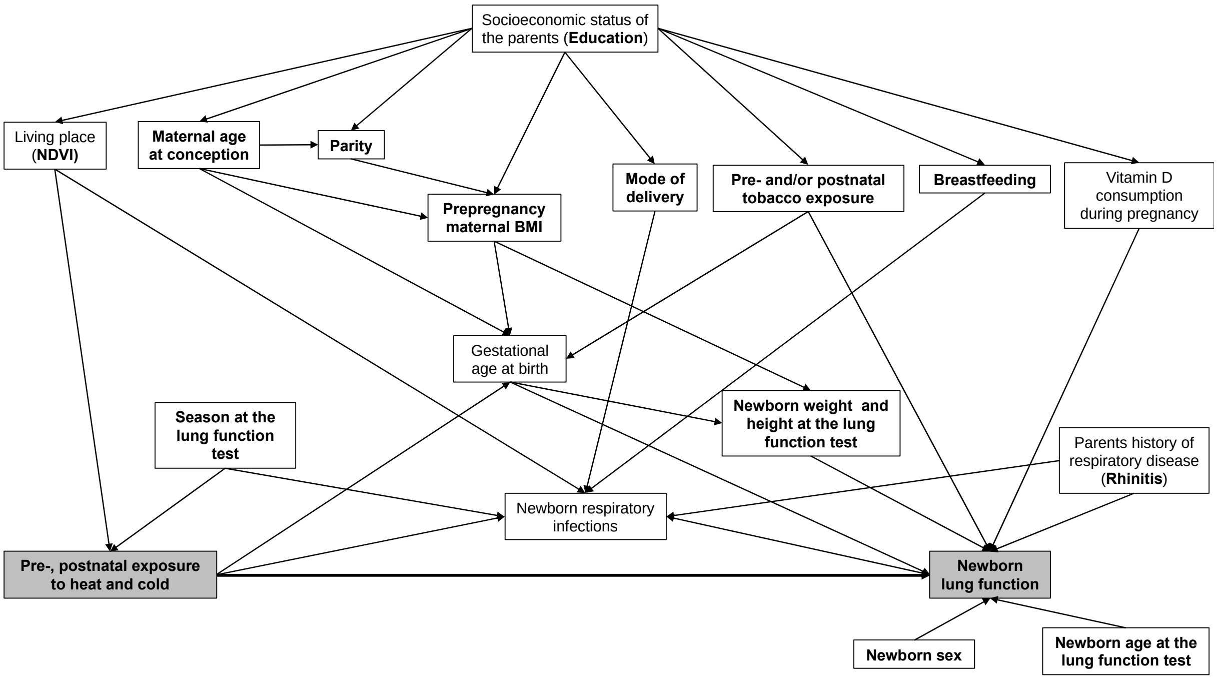

Directed acyclic graph describing the relationship between pre-, postnatal exposure to heat and cold and newborn lung at two months, in the framework of the SEPAGES cohort
*In **bold**: Exposure, outcome of interest and covariates included in the main analyses of the present study.*

Statistical analysis

The relationships between exposure to ambient temperature and newborn lung function measurements (continuous variables) were investigated using Distributed Lag Non-linear Models (DLNM)[7].

For each lung function measurement and each exposure indicator, two time periods of interest were examined simultaneously: 1) chronic exposure, in weeks, including the first 35 gestational weeks and first 4 weeks after delivery; 2) acute exposure, in days, encompassing the 7 days preceding/including the day of the lung function tests. This strategy was adopted because the DLNM require exposure matrices of the same length for all participants. Yet not all births occurred at 40 weeks' gestation and not all respiratory measurements were carried out at the same age exactly. Therefore, we discarded the time period after 35 weeks and before delivery and stopped at 4 weeks after delivery for chronic exposure. This strategy also allowed to clearly distinguish between prenatal and postnatal exposure. Besides, stopping at 4 weeks after delivery avoided an overlap between the chronic and acute exposure matrices.

For each lung function indicator, two kinds of models were fitted: 1) For Tmin, Tmean, Tmax, a first set of models including both chronic (1st cross-basis, weekly average) and acute (2nd cross-basis, daily values) exposure to temperature; 2) For Tsd, a second set of models including chronic exposure (1st cross-basis, weekly SD) and adjusting for acute exposure (single value per participant) to temperature variability over 7 days.

We hypothesized the relationships between temperature exposure (acute and chronic) and lung function measurements were non-linear and modelled the dose-response and lag-response using natural cubic splines. The number of degrees of freedom was set to 2 for all splines, based on the Akaike information criterion and parsimony. The lag-response (β and 95% confidence interval (95%CI)) curves were modelled for heat and cold (95th, 5th temperature percentiles, respectively; thresholds based on previous research works[7–10] and considering population size) exposure compared to the median exposure. Critical windows were identified and defined based on the plots illustrating the lag-specific effect of cold and heat on the various lung function measurements. A time period was considered as a relevant critical window as long as the lag-specific effect and it is 95% confidence interval did not cross β = 0. We report both the cumulative risk associated with exposure throughout the entire duration of a critical window and some illustrations of the risk associated with a single week of exposure.

Sensitivity analyses on LCI and FRC were performed excluding newborns showing high hypoventilation levels (higher than the 75th percentile).

Analyses were carried out using the R © (version 4.0.4) software and the dlnm package (version 2.4.7).

© 2023 Guilbert A et al. *JAMA Network Open.*

eTable 1: Comparison of the included and excluded participants

Characteristics	Included (n = 343)	Excluded (n = 141)	Pvalue[b]
	n (%)		
Highest level of parental education			0.11
Less than a Master's degree	97 (28)	51 (36)	
Master's degree or more	246 (72)	90 (64)	
Parental rhinitis			0.61
No	125 (36)	33 (33)	
Yes	218 (64)	67 (67)	
Missing	0	41 (29)	
Pre- and/or postnatal tobacco exposure			0.82
No	251 (73)	92 (75)	
Yes	92 (27)	31 (25)	
Missing	0	18 (13)	
Mode of delivery			0.14
Vaginal	291 (85)	107 (79)	
Caesarean	52 (15)	29 (21)	
Missing	0	5 (4)	
Parity			0.17
No child	150 (44)	72 (51)	
One child or more	193 (56)	69 (49)	
Breastfeeding			0.080
Not breastfed at two months	46 (13)	23 (21)	
Still breastfed at two months	297 (87)	87 (79)	
Missing	0	31 (22)	
Newborn gender			0.86
Boys	183 (53)	75 (55)	
Girls	160 (47)	62 (45)	
Missing	0	4 (3)	
Season at the lung function test			0.69
Winter	86 (25)	17 (19)	
Spring	75 (22)	22 (25)	
Summer	74 (22)	21 (24)	
Fall	108 (31)	29 (33)	
Missing	0	52 (37)	
	Median (IQR)		
Mother's age at conception (Years)	32.3 (30.0, 35.2)	31.7 (29.8, 34.9)	0.58
Mother's BMI before pregnancy (kg/m²)	21.4 (19.7, 24.0)	21.8 (19.8, 23.8)	0.45
Missing	0	4 (3)	
Child age at the lung function test (weeks)	6.7 (6.3, 7.3)	7.3 (6.6, 8.1)	<0.001
Missing	0	52 (37)	
Child height at the lung function test (cm)	56.3 (55.0, 58.0)	56.3 (55.0, 58.0)	0.99
Missing	0	53 (38)	
Child weight at the lung function test (kg)	4.8 (4.5, 5.2)	4.8 (4.4, 5.2)	0.90
Missing	0	52 (37%)	
Summer NDVI the year of birth	0.5 (0.3, 0.6)	0.5 (0.4, 0.6)	0.88
Missing	0	4 (3)	
Night-time temperature (Tmin; °C)[a]	7.8 (6.0, 9.4)	7.5 (5.9, 9.4)	0.61
Missing	0	4 (3)	
Overall temperature (Tmean; °C)[a]	12.7 (10.5, 14.5)	12.0 (10.4, 14.3)	0.45
Missing	0	4 (3)	
Daytime temperature (Tmax; °C)[a]	18.6 (16.1, 20.8)	17.8 (15.9, 20.4)	0.43
Missing	0	4 (3)	
Variability of mean temperature (Tsd)[a]	7.2 (6.5, 7.8)	7.1 (6.4, 7.8)	0.55
Missing	0	4 (3)	

Abbreviations: BMI: Body Mass Index; IQR: InterQuartile Range; NDVI: Normalized Difference Vegetation Index; SD: Standard Deviation.
[a] Average over the pregnancy.
[b] Chi test for categorical variables and Wilcoxon test for continuous variables.
n = 484 mother-child pairs from the SEPAGES cohort.

© 2023 Guilbert A et al. *JAMA Network Open.*

eTable 2: Cumulative change in lung function measurements significantly associated with exposure to cold, heat or variability of temperature

	Measurement	Exposure	Window	β (95%CI)
Whole population (n = 343)		**Cold (5th pct vs. median)**		
	FRC	Tmin (-2°C vs 7°C)	Days 2-3 before the lung function tests	2.1 (0.1; 4.1)
		Tmean (1°C vs 12°C)	Days 1-3 before the lung function tests	5.4 (0.9; 9.9)
	Respiratory rate	Tmin (-2°C vs 7°C)	Gestational weeks 1-25	18.1 (4.1; 32.1)
		Tmean (1°C vs 12°C)	Gestational weeks 1-15	12.0 (0.8; 23.1)
		Tmax (5°C vs 18°C)	Gestational weeks 7-12	4.1 (0.1; 8.2)
		**Heat (95th pct vs. median)**		
	LCI	Tmin (17°C vs 7°C)	Gestational weeks 1-4	0.3 (0.0; 0.6)
		Tmean (24°C vs 12°C)	Gestational weeks 1-13	1.0 (0.1; 1.9)
		Tmax (32°C vs 18°C)	Gestational weeks 1-6	0.5 (0.0; 0.9)
	FRC	Tmin (17°C vs 7°C)	Gestational weeks 25-35 & weeks 0-4 after delivery	-17.2 (-31.0; -3.4)
		Tmean (24°C vs 12°C)	Gestational weeks 20-35 & weeks 0-4 after delivery	-26.3 (-46.4; -6.2)
		Tmean (24°C vs 12°C)	Days 2-3 before the lung function tests	-3.2 (-6.1; -0.3)
		Tmax (32°C vs 18°C)	Gestational weeks 24-35 & weeks 0-4 after delivery	-18.4 (-34.2; -2.7)
		Tmax (32°C vs 18°C)	Days 2-3 before the lung function tests	-3.2 (-6.0; -0.5)
		**Low variability (5th pct vs. median)**		
	FRC	Tsd (1°C vs 2°C)	Gestational weeks 7-18	10.4 (0.7; 20.1)
		**High variability (95th pct vs. median)**		
	FRC	Tsd (4°C vs 2°C)	Gestational weeks 10-35 & weeks 0-4 after delivery	47.9 (20.6; 75.3)
Males (n = 183)		**Cold (5th pct vs. median)**		
	Tidal volume	Tmin (-2°C vs 7°C)	Days 2-3 before the lung function tests	1.2 (0.1; 2.2)
		Tmean (1°C vs 12°C)	Gestational weeks 26-35 & weeks 0-4 after delivery	9.7 (0.5; 18.9)
		Tmean (1°C vs 12°C)	Days 2-3 before the lung function tests	1.5 (0.3; 2.7)
	Respiratory rate	Tmin (-2°C vs 7°C)	Days 2-3 before the lung function tests	-1.7 (-3.4; -0.1)
		**Heat (95th pct vs. median)**		
	FRC	Tmean (24°C vs 12°C)	Days 2-3 before the lung function tests	-4.7 (-9.2; -0.2)
		Tmax (32°C vs 18°C)	Days 2-4 before the lung function tests	-7.5 (-13.0; -2.0)
	Tidal volume	Tmean (24°C vs 12°C)	Gestational weeks 1-23 after conception	14.6 (2.1; 27.0)
	Respiratory rate	Tmax (32°C vs 18°C)	Gestational weeks 9-16 after conception	-6.1 (-12.2; -0.1)
		**High variability (95th pct vs. median)**		
	FRC	Tsd (4°C vs 2°C)	Weeks 23-31 after conception	13.8 (0.7; 26.9)

© 2023 Guilbert A et al. *JAMA Network Open.*

	Measurement	Exposure	Window	β (95%CI)
	Minute ventilation	Tsd (4°C vs 2°C)	Gestational weeks 26-35 & weeks 0-4 after delivery	483.6 (78.6; 888.6)
	Respiratory rate	Tsd (4°C vs 2°C)	Gestational weeks 30-35 & weeks 0-4 after delivery	14.4 (1.4; 27.4)
Females (n = 160)		**Cold (5th pct vs. median)**		
	FRC	Tmin (-2°C vs 7°C)	Gestational weeks 9-35 & weeks 0-4 after delivery	-55.3 (-91.8; -18.7)
		Tmean (1°C vs 12°C)	Gestational weeks 15-29	-21.9 (-42.4; -1.3)
		Tmean (1°C vs 12°C)	Days 2-3 before the lung function tests	3.4 (0.1; 6.7)
		Tmax (5°C vs 18°C)	Gestational weeks 13-20	-9.5 (-18.9; -0.1)
	Tidal volume	Tmin (-2°C vs 7°C)	Gestational weeks 12-35 & weeks 0-4 after delivery	-25.6 (-41.4; -9.7)
		Tmean (1°C vs 12°C)	Gestational weeks 14-35 & weeks 0-4 after delivery	-23.8 (-43.1; -4.4)
		Tmax (5°C vs 18°C)	Gestational weeks 17-35 & weeks 0-4 after delivery	-23.0 (-42.0; -4.1)
	Respiratory rate	Tmin (-2°C vs 7°C)	Gestational weeks 5-35 & weeks 0-4 after delivery	52.6 (20.2; 85.0)
		Tmean (1°C vs 12°C)	Gestational weeks 6-35 & weeks 0-1 after delivery	45.5 (10.1; 81.0)
		Tmax (5°C vs 18°C)	Gestational weeks 8-32	33.8 (5.0; 62.6)
	tPTEF/tE ratio	Tmin (-2°C vs 7°C)	Days 0-2 before the lung function tests	6.0 (0.3; 11.7)
		Tmean (1°C vs 12°C)	Days 0-1 before the lung function tests	5.3 (0.0; 10.5)
		Tmax (5°C vs 18°C)	Days 0-2 before the lung function tests	6.7 (0.0; 13.4)
		**Heat (95th pct vs. median)**		
	FRC	Tmin (17°C vs 7°C)	Gestational weeks 21-35 & weeks 0-4 after delivery	-35.4 (-58.6; -12.2)
		Tmean (24°C vs 12°C)	Gestational weeks 20-35 & weeks 0-4 after delivery	-39.7 (-68.6; -10.7)
		Tmax (32°C vs 18°C)	Gestational weeks 26-35 & weeks 0-4 after delivery	-23.2 (-43.8; -2.5)
	Tidal volume	Tmin (17°C vs 7°C)	Gestational weeks 29-35 & weeks 0-4 after delivery	-7.0 (-13.6; -0.4)
	Minute ventilation	Tmax (32°C vs 18°C)	Gestational weeks 2-21	522.6 (59.9; 985.3)
	Respiratory rate	Tmin (17°C vs 7°C)	Gestational weeks 16-35 & weeks 0-4 after delivery	29.3 (7.6; 51.0)
		Tmean (24°C vs 12°C)	Gestational weeks 14-35 & weeks 0-1 after delivery	28.0 (4.2; 51.9)
		Tmax (32°C vs 18°C)	Gestational weeks 14-35 & weeks 0-3 after delivery	30.9 (5.3; 56.5)
		**Low variability (5th pct vs. median)**		
	LCI	Tsd (1°C vs 2°C)	Gestational weeks 12-30	1.6 (0.3; 3.0)
		**High variability (95th pct vs. median)**		
	FRC	Tsd (4°C vs 2°C)	Gestational weeks 8-35 & weeks 0-1 after delivery	57.1 (21.1; 93.2)

Abbreviations: BMI: Body Mass Index; CI: Confidence Interval; FRC: Functional Residual Capacity; LCI: Lung Clearance Index; NDVI: Normalized Difference Vegetation Index; pct: Percentile; SD: Standard Deviation.
Models adjusted for maternal age at conception, highest level of education of the parents, prepregnancy maternal BMI, parity, parents' rhinitis, mode of delivery, breastfeeding, pre- and/or postnatal tobacco exposure, child sex, child age, child weight and height at the lung function test, season at lung function test, NDVI the year of birth.
The number of associations presented varies across sex because the table restricts to the statistically significant windows (i.e. significant associations are not consistently observed between heat, cold and each respiratory measurement for both males and females). The critical window positions can also vary according to the sex.
n = 343 mother-child pairs from the SEPAGES cohort.

© 2023 Guilbert A et al. *JAMA Network Open.*

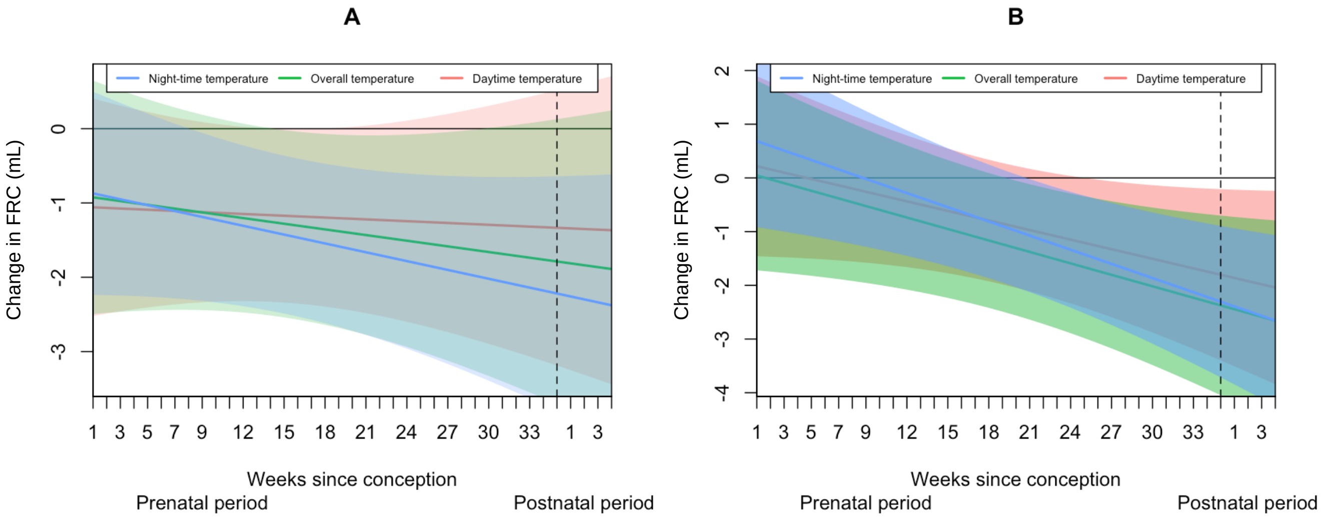

eFigure 1: Lag-specific effect of cold (A) and heat (B) compared with the median temperature on functional residual capacity for females

Shaded areas represent the 95% CI of the estimate.
Abbreviations: CI: Confidence Interval.
Models adjusted for maternal age at conception, highest level of education of the parents, prepregnancy maternal BMI, parity, parents' rhinitis, mode of delivery, breastfeeding, pre- and/or postnatal tobacco exposure, child sex, child age, child weight and height at the lung function test, season at lung function test, NDVI the year of birth.
n = 343 mother-child pairs from the SEPAGES cohort.

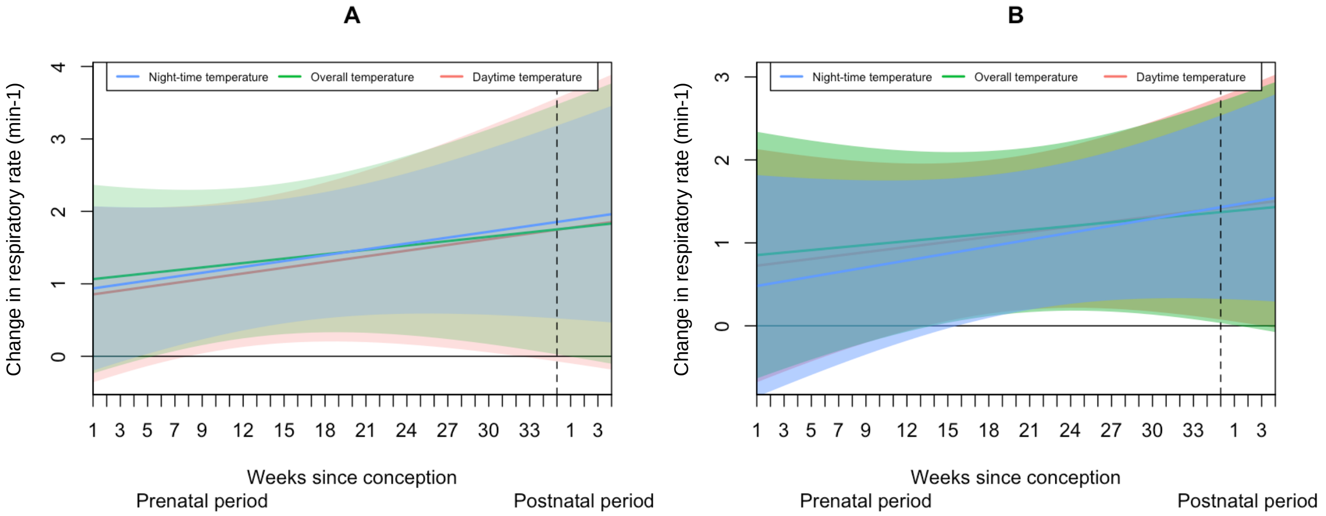

eFigure 2: Lag-specific effect of cold (A) and heat (B) compared with the median temperature on respiratory rate for females

Shaded areas represent the 95% CI of the estimate.
Abbreviations: CI: Confidence Interval.
Models adjusted for maternal age at conception, highest level of education of the parents, prepregnancy maternal BMI, parity, parents' rhinitis, mode of delivery, breastfeeding, pre- and/or postnatal tobacco exposure, child sex, child age, child weight and height at the lung function test, season at lung function test, NDVI the year of birth.
n = 343 mother-child pairs from the SEPAGES cohort.

© 2023 Guilbert A et al. *JAMA Network Open.*

eReferences

1. Hough I, Just AC, Zhou B, Dorman M, Lepeule J, Kloog I. A multi-resolution air temperature model for France from MODIS and Landsat thermal data. *Environ Res*. 2020;183:109244. doi:10.1016/j.envres.2020.109244
2. Bates JH, Schmalisch G, Filbrun D, Stocks J. Tidal breath analysis for infant pulmonary function testing. ERS/ATS Task Force on Standards for Infant Respiratory Function Testing. European Respiratory Society/American Thoracic Society. *Eur Respir J*. 2000;16(6):1180-1192. doi:10.1034/j.1399-3003.2000.16f26.x
3. Gustafsson PM, Bengtsson L, Lindblad A, Robinson PD. The effect of inert gas choice on multiple breath washout in healthy infants: differences in lung function outcomes and breathing pattern. *J Appl Physiol Bethesda Md 1985*. 2017;123(6):1545-1554. doi:10.1152/japplphysiol.00524.2017
4. Mortamais M, Chevrier C, Philippat C, et al. Correcting for the influence of sampling conditions on biomarkers of exposure to phenols and phthalates: a 2-step standardization method based on regression residuals. *Environ Health Glob Access Sci Source*. 2012;11:29. doi:10.1186/1476-069X-11-29
5. Robinson NP, Allred BW, Jones MO, et al. A Dynamic Landsat Derived Normalized Difference Vegetation Index (NDVI) Product for the Conterminous United States. *Remote Sens*. 2017;9(8):863. doi:10.3390/rs9080863
6. Lykkedegn S, Sorensen GL, Beck-Nielsen SS, Christesen HT. The impact of vitamin D on fetal and neonatal lung maturation. A systematic review. *Am J Physiol Lung Cell Mol Physiol*. 2015;308(7):L587-602. doi:10.1152/ajplung.00117.2014
7. Gasparrini A, Armstrong B, Kenward MG. Distributed lag non-linear models. *Stat Med*. 2010;29(21):2224-2234. doi:10.1002/sim.3940
8. Vicedo-Cabrera AM, Sera F, Guo Y, et al. A multi-country analysis on potential adaptive mechanisms to cold and heat in a changing climate. *Environ Int*. 2018;111:239-246. doi:10.1016/j.envint.2017.11.006
9. Barnett AG, Hajat S, Gasparrini A, Rocklöv J. Cold and heat waves in the United States. *Environ Res*. 2012;112:218-224. doi:10.1016/j.envres.2011.12.010
10. Pascal M, Wagner V, Corso M, Laaidi K, Ung A, Beaudeau P. Heat and cold related-mortality in 18 French cities. *Environ Int*. 2018;121:189-198. doi:10.1016/j.envint.2018.08.049

© 2023 Guilbert A et al. *JAMA Network Open.*
