# Supplementary material for: Association of Prenatal and Postnatal Exposures to Warm or Cold Air Temperatures With Lung Function in Young Infants
Source: JAMA Netw Open. 2023 Mar 17;6(3):e233376. doi: 10.1001/jamanetworkopen.2023.3376 (PMC10024202; doi:10.1001/jamanetworkopen.2023.3376)
Supplement: Supplement 2. — Data Sharing Statement [file jamanetwopen-e233376-s002.pdf]

Data Sharing Statement

Guilbert. Association of Prenatal and Postnatal Exposures to Warm or Cold Air Temperatures With Lung Function in Young Infants. *JAMA Netw Open*. Published March 17, 2023. doi:10.1001/jamanetworkopen.2023.3376

Data
Data available: Yes
Data types: Deidentified participant data, Data dictionary
How to access data: The SEPAGES datasets analyzed in the presented study are not publicly available as they are containing information that could compromise the research participant's privacy/consent. However, they are available on reasonable request and with permission from the SEPAGES Steering Committee by contacting the SEPAGES team by email: contact-sepages@inserm.fr.
When available: With publication

Supporting Documents
Document types: None

Additional Information
Who can access the data: Researchers whose proposed use of the data has been approved by the SEPAGES Steering Committee
Types of analyses: For a specified purpose
Mechanisms of data availability: With investigator support, after approval of a proposal and with a signed data access agreement
